# The Effects of Aerobic and Resistance Exercises on the Cognitive and Physical Function of Persons with Mild Dementia: A Randomized Controlled Trial Protocol

**DOI:** 10.3390/healthcare11050677

**Published:** 2023-02-25

**Authors:** Vasileios Papatsimpas, Sotiria Vrouva, Marianna Papadopoulou, George Papathanasiou, Daphne Bakalidou

**Affiliations:** 1 Physiotherapy Department, School of Health and Care Sciences, University of West Attica (UNIWA), 12243 Athens, Greece; 2 Laboratory of Neuromuscular and Cardiovascular Study of Motion (LANECASM), School of Health and Care Sciences, University of West Attica (UNIWA), 12243 Athens, Greece; 3 Department of Physical Therapy, General Hospital of Athens G. GENNIMATAS, 11527 Athens, Greece; 4 Department of Physical Therapy, 401 Army General Hospital of Athens, 11525 Athens, Greece

**Keywords:** dementia, Alzheimer’s disease, aerobic exercise, resistance exercise, functionality, cognitive function

## Abstract

Dementia causes deterioration in cognitive and physical functions. The scope of this study is to investigate the effect of different exercise programs on cognitive functions and functionality of persons suffering from mild Alzheimer’s disease (AD) by generating information on the exercise types and their parameters. A randomized controlled trial (RCT) will be performed involving aerobic and resistance exercise interventions, taking place both at the sample collection center and at home. Participants will be randomly divided into two different intervention groups and a control group. All groups will be assessed twice; once at baseline and once after 12 weeks. The primary outcome shall comprise the effect of exercise programs on cognitive functions using cognitive testing, such as Addenbrooke’s Cognitive Examination—Revisited (ACE-R), Mini Mental State Examination (MMSE), Trail Making Test A-Β (TMT A-B), and Digit Span Test (DST): Digit Span Forward (DSF) and Digit Span Backward (DSB). The effect on functionality will be assessed using the Senior Fitness Test (SFT), Berg Balance Scale (BBS), and Instrumental Activities of Daily Living Scale (IADL) questionnaire. Secondary outcomes include the effect of exercise on depression using the Geriatric Depression Scale-15 (GDS-15), on physical activity using the International Physical Activity Questionnaire (IPAQ), as well as the participants’ compliance with the intervention. This study will investigate the possible effect of intervention of different exercise types and the comparison between them. Exercise forms a low-cost and reduced-risk intervention.

## 1. Introduction

Aging, as an inevitable fact of human life, is a dynamic and evolving process involving morphological, functional, biochemical, and psychological changes [1]. It is estimated that between 2015 and 2050, the number of people over 60 years old will have increased from 900 million to 2 billion globally [1,2]. As the number of elderly people increases worldwide, so does the number of people suffering from dementia, which poses a serious epidemic problem to humanity, whilst becoming a major social and public health issue [2,3,4,5].

There are several types of dementia related to different causes (changes in the brain) such as Alzheimer’s disease (accumulation of the abnormal proteins beta-amyloid and phosphorylated tau, as well as the degeneration of nerve cells), vascular dementia (vascular pathology), frontotemporal dementia (frontotemporal lobar degeneration), dementia with Lewy bodies, and mixed types [5,6]. Although dementia is common in the elderly, it is not a normal part of aging [5]. There is a small percentage, around 10%, in which dementia is detected in middle age and is due to so-called early-onset Alzheimer’s disease [6]. Initially, there is episodic loss of short-term memory, then gradually, the memory is lost completely [6]. Judgment disorder, disorientation, and language disorder follow [6]. As the disease progresses, the decline in cognitive and physical function often accompanies depression, apathy, agitation, psychosis, sleep disorders, as well as extrapyramidal symptoms [6].

There have been multi-domain lifestyle interventions to reduce and prevent dementia risk, which include pharmacological and non-pharmacological treatments such as exercise, Mediterranean diet, physical activity, etc. [7].

In recent years, there has been a particular interest in the role of physical exercise as prevention as well as a therapeutic strategy for the management of persons suffering from dementia [8,9]. Exercise has been shown to have positive effects on functionality, fall prevention and reduction, cognition, sleep disturbances, mood, and quality of life in dementia patients [10]. Usually, the recommendation for exercise in patients with AD is made without specifying types, frequency, or intensity of exercise [10,11]. However, both the exercise burden and the effect on the patient vary significantly from person to person [10].

The benefits of aerobic exercise have been demonstrated in several studies, although we do not have enough studies on the benefits of resistance exercise in people with dementia [1,9,11].

The purpose of this study is to investigate the effect of exercise on the cognitive and physical functions in patients with Alzheimer’s disease and to provide information on appropriate types of exercise and its parameters in order to maintain and improve cognition and physical functions.

## 2. Material and Methods

### 2.1. Source of Data

The present research is a double-blind, randomized prospective intervention study on the effect of exercise on cognitive functions and functionality in persons with dementia. This protocol describes a 12-week randomized clinical trial control applied to patients with mild dementia (Alzheimer’s Disease). The main hypothesis of the study aims to establish not only the effect of exercise on the cognitive and physical function of patients but also the magnitude of that effect on cognitive and physical functioning in relation to the various exercise protocols.

### 2.2. Participants and Eligibility

The sample of the study population will be selected by the method of purposive sampling, observing the rules of appropriateness and adequacy.

In order to estimate the sample of patients required for any statistical relation to be established as significant, we perform a priori power analysis using the software GPower 3.1. The parameters applied included: (a) alpha level of 0.05 (b) statistical power at least of >80%, (c) the effect size or else the magnitude of the effect of the exercise is expected to be small to moderate and was determined (f = 0.23–0.24). The effect size (ES) is estimated based on previous studies on the cognitive effects of combined aerobic and strength training and single aerobic training using a similar intervention [12,13,14]. Since we are going to study multiple outcomes (cognitive function and functionality), we used the weakest exercise outcome association for sample size calculation. With the above parameters and the fact that the present study provides information for three groups, the calculator showed us that by using 171–186 participants in total, or 57–62 individuals per group, we could secure a meaningful statistical power 80% for all the assessments performed.

Participants will be recruited with the assistance of the Amarousion Day Center of the Alzheimer Society of Athens as well as from the outpatient clinic of the Athens General Hospital “G. Gennimatas”. Potential study participants are to be notified by the center’s physician. The participants will be persons with mild Alzheimer’s disease which was determined in accordance with the diagnosis (DSM-IV and NINCDS/ADRDA diagnostic criteria) [15,16].

All study participants will receive full and clear information, both verbally and in writing, explaining the purpose of the study, data confidentiality and the voluntary nature of participation without financial incentive. They will also be informed that they can withdraw their participation from the research at any time. Whilst each question will be explained, the participants will be able to call the main researcher anonymously at all times, whenever they are in need of more information or clarifications. When the study is completed, the participants will be informed on the results of the study, if they so wish. As soon as all patients have been fully informed, they will be requested to provide written consent of participation in this study. The preparation costs and relevant expenses shall be borne by the researchers.

### 2.3. Participant Inclusion and Exclusion Criteria

Participants meeting the following criteria will be able to participate in the research: Age ≥ 65, diagnosis of mild AD with MMSE score: 20–24/30, without hearing and/or vision impairment which might complicate their participation. The participant must be able to walk to the intervention place, have a caregiver, as well as medical consent to participate in the exercise routine. The participant must not currently participate in any other exercise program, be on stable medication for at least 2 months, and must possess ability of consent according to the treating physician and the treatment team.

Participants will be excluded if they suffer from other neurological disease and severe diseases which would not allow participation in exercise (severe psychiatric illnesses, uncontrolled arterial hypertension, severe cardiorespiratory problems, severe musculoskeletal problems), cancer, surgery during the previous year, severe vision and/or hearing problems, or alcohol and/or drug abuse.

### 2.4. Randomization and Blinding

The randomization will be performed by an independent researcher, who will remain hidden from the rest of the research team involved in the interventions, the assessments, the collection of outcome measurements, and the analysis of data. Prior to the baseline assessment, the participants are to be randomly assigned to one of three groups, A, B, and C (two exercise intervention groups and one usual care—control group), by using random number generator. The allocation sequence will be concealed from the researchers related to the study and the intervention will be assigned using sealed envelopes based on order of recruitment. The random allocation will not appear on the assessment forms and the participants’ names will be replaced by code numbers. 

All participants will be assessed by means of two evaluations prior to the start of the intervention and immediately after the completion of the program (12 weeks) with an optional 12-week extension. Professionals participating in the intervention program will not participate in the physical assessment.

During the assessment, in addition to the demographic data, the following data will be collected: somatometric characteristics (height–weight–body mass index), medication, serious illnesses, any incidents involving falling during the last six months, arterial pressure, heart pulses, oxygen saturation, smoking habits, and level of education. Demographics will only be recorded during the initial assessment.

**Figure 1:**
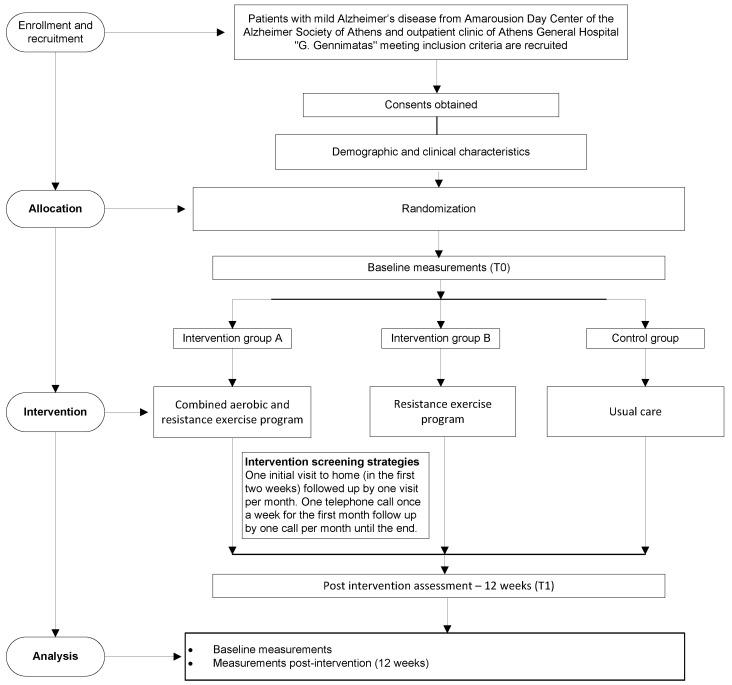
Study flowchart.

During each assessment, participants and examiners are not allowed to provide or request any information regarding the exercise program being performed. Furthermore, the assessors will be blinded to previous test results. All the above will be included in the study flowchart (Figure 1) in accordance with the CONSORT guidelines.

## 3. Outcome Measures and Measurement Procedures

The assessment of physical functions will be carried out by two physiotherapists experienced in the management of patients with dementia, whilst the neuropsychological evaluation will be performed by a neuropsychologist or psychologist of the center. All assessors who will participate in data collection will be trained in study procedures prior to the study.

The primary objective of the study is to determine the effects of exercise on the cognitive function and functionality of persons suffering from mild Alzheimer’s disease. Addenbrooke’s Cognitive Examination—Revisited (ACE-R), Mini Mental State Examination (MMSE), Trail Making Test A (TMT-A), Trail Making Test B (TMT-B), Digit Span Test Forward and Backward (DST F-B) were selected for the evaluation of cognitive function. The Senior Fitness Test (SFT), Berg Balance Scale (BBS), and Instrumental Activities of Daily Living Scale (IADL) were selected for the functionality evaluation.

The secondary aim of the study is to determine the effect of exercise on an individual’s mood, for which the Geriatric Depression Scale-15 (GDS-15) was chosen, as well as the effect of exercise on physical activity by using the International Physical Activity Questionnaire (IPA-Q) short edition—7 items, as well as compliance with the intervention.

All assessment tools have been proven valid and reliable in Greek and have been widely used in studies involving elderly and dementia patients [17,18,19,20,21,22,23,24,25,26,27].

### 3.1. Description of Outcome Measures

#### 3.1.1. Assessment of Cognitive Functioning and Mood

##### Addenbrooke’s Cognitive Examination Revised

The ACE-R is a brief cognitive assessment tool [17]. The ACE-R takes between 12 and 20 min (16 on average) [28]. It contains five subscales, each of which represents a cognitive domain: (a) attention/orientation (18 points), (b) memory (26 points), (c) verbal fluency (14 points), (d) language (26 points), and (e) visuospatial ability (16 points) [17,28]. The maximum ACE-R score is 100, consisting of the addition of all domains indicating better cognitive function [17,28].

##### Mini Mental State Examination

The MMSE is the most widely used assessment tool for diagnosing cognitive disorders. Its administration procedure is simple and takes 5 to 10 min [18,29]. The MMSE consists of tests (30 sub questions) which examine five cognitive domains: (a) orientation, (b) attention–concentration, (c) memory, (d) language, and (e) visuospatial ability [30]. For each correct answer, we obtain one point, and for each wrong one, zero points, with a maximum performance in the test of 30 points [29,30,31]. Higher performance indicates better cognitive functioning [29,30,31].

##### Trail Making Test A–B

The TMT is an easy and quick neuropsychological test; it assesses cognitive abilities such as attention, processing speed, and executive functions [32]. The TMT consists of two parts in which the examinee is instructed to connect a set of 25 dots as quickly as possible while maintaining accuracy [19,32]. In the first part (TMT-A), the targets are all numbers (1, 2, 3, etc.) and the examinee must connect them in consecutive order [19,32]. In the second part (TMT-B), the subject alternates between numbers and letters (1, A, 2, B, etc.) [19,32]. The aim of the test is for the assessee to complete both parts as quickly as possible [19]. The test of each part ends after 5 min even if it is not completed [32].

##### Digit Span test

One of the most common tests for assessing attention and working memory is the Digit Span Test [33,34]. With the DST, recent memory is assessed [34]. The score is the sum of the number of digits from the forward repetition in which the examinee is asked to repeat after the examiner (DST-F) and the number of digits from the reverse repetition (DST-B) [33,34]. The test is terminated when the subject does not accurately record the trial in a sequence length or when the maximum list length is reached [33,34]. The total number of subtests (DST F-B) are summed to produce a total score [33]. Scoring is conducted with two points if the examinee repeats both series correctly at the particular difficulty level under consideration, with one point if they repeat one series correctly, and with zero points if they fail both [34].

##### Geriatric Depression Scale-15

The GDS-15 is a short-form self-report test of 15 questions [25,35,36,37]. The GDS-15 is a widely used scale internationally [25]. The 15 questions of the short form of the scale require only a YES–NO answer and take approximately 5–7 min [25]. The questionnaire is completed with an interview [35,36,37]. Each answer of the test is scored 0 or 1 [25,38]. In questions 1, 5, 7, 11, 13, the answer “NO” is scored with 1 point, while in the remaining questions, the answer “YES” is scored with 1 point [25,38]. For the severity of depression, the following categorization is followed: 0–5 points correspond to “absence of depressive symptoms”, 6–10 to “moderate depression”, and 11–15 to “severe depression” [25,38].

#### 3.1.2. Functional Fitness Assessment

##### Senior Fitness Test

The SFT is a practical set of tests for clinical use and is suitable for both healthy seniors and people with dementia and is simple to use [39,40]. It is an international easy-to-use tool for measuring functional fitness in older people with or without cognitive impairment and is suitable for both research and clinical purposes [21,39,40,41]. It is simple to use, requires no expensive tools or technical expertise, and can be performed in any location [40,42,43,44,45]. The test includes six functional tests of arm and leg strength, endurance, balance, agility, and flexibility and takes approximately 30–40 min to complete [41,42,43]. The Senior Fitness Test was modified and adapted to Greek data. During the Arm Curl test, weights of 2 kg and 3.5 kg will be used, for women and men, respectively, and flexibility will be measured in centimeters [41,44,45,46,47].

##### Berg Balance Scale

The BBS is a popular and well-established clinical tool for assessing balance [22]. It is known as a balance measurement tool in the elderly but has also been tested for its reliability and validity in assessing balance in patients with various neurological diseases with very good results [22]. The BBS assesses the subjects’ static and dynamic balance ability and predicts the likelihood of falls by performing simple tests that require good balance [22,48]. It consists of 14 simple tests, which are often performed in daily activities and is mainly used to assess people with mobility difficulties such as the elderly [48]. The time required to complete all tests is 15–20 min [22,49]. Test takers are scored on a scale of 0–4 based on their ability to perform the required tests [22,49]. The total score ranges from 0 to 56 and a score of less than 45 equates to an increased risk of falls [22,49]. Higher scores indicate better performance and greater independence [22,49]. A cut-off point of 45/56 has been suggested for independent and safe movement [22,49]. It is an easy and quick physical performance test that requires no training or special equipment [22].

##### Instrumental Activities of Daily Living Scale

The IADL was first presented in 1969 by Lawton and Brody [50]. The IADL questionnaire explores individuals’ basic ability to care for themselves and refers to higher levels of performance [23,24,50,51]. The present questionnaire is a suitable tool for assessing independent living skills in both healthy elderly and elderly patients with dementia and mild cognitive impairment [23,24,50,51,52]. The questionnaire is completed with an interview [51]. The IADL is an assessment tool, easy to administer, and administration time is 10–15 min [51]. The IADL questionnaire scale measures eight domains of functioning: telephone use, shopping, food preparation, housekeeping, laundry, mode of transportation, responsibility for personal medication, and ability to manage finances [23,50,51]. Women are rated in all 8 functional areas [23]. Men are excluded from the areas of food preparation, cleaning, and laundry [23]. Each domain is scored 0 or 1, respectively, when respondents report difficulty, or receive help with the task, or could not perform the task [23,50,51]. The total score ranges from 0 (low functioning, dependent) to 8 (high functioning, independent) for women and 0 to 5 for men [23,50,51].

##### International Physical Activity Questionnaire (Short Edition—7 Items)

The IPAQ was developed as a means to monitor and assess physical activity and inactivity for all ages, including the elderly [53,54,55]. In our study, we will use the IPAQ—GR (short version—7 items) which consists of seven questions which participants are asked to answer in order to determine the number of days and minutes per day they spend on either vigorous or moderate physical activity, how much time they spent walking over the past seven days as well as sitting down during a typical week [26,27]. The questionnaire is completed by means of an interview [26]. The activity withdrawal period concerns the previous seven days [26,27].

According to IPAQ scoring, the physical activity is categorized as follows: (a) low physical activity, (b) moderate physical activity, and (c) high physical activity [26].

## 4. Therapeutic Intervention

The intervention will include aerobic as well as resistance exercise. For this purpose, three groups will be created, two (2) intervention groups and one (1) control group. The first intervention group will follow a program containing both aerobic and resistance exercise and the second group will follow a program consisting only of resistance exercise. The intervention will be composed of a different duration and frequency for each type of exercise but with moderate intensity for both groups.

The intervention was planned pursuant to studies and guidelines from the American Sports Medicine Association (ACSM) in respect of exercise and prescription, the American Society of Cardiology (AHA), and in accordance with the recommendations of the World Health Organization (WHO) for physical activity in older adults [4,14,56,57,58,59,60,61,62].

Physiotherapists delivering the exercise intervention will receive two days of program-specific training by the principal investigator physiotherapist prior to the start of the program.

During the intervention period, patients belonging to both intervention groups will not be allowed to participate in any other exercise program. At the same time, patients in the control group will not be allowed to participate in any other form of intervention (exercise program) other than that provided in routine care.

Caregivers will have an essential role in supervising exercise sessions to ensure adherence to guidelines. Caregivers will receive training for this role. Caregiver supervision will include both walking with the participant and supervision of resistance exercise to be performed at home as well as supervision of diary keeping. The intervention will include:

**Intervention Group A:** Combined aerobic and resistance exercise program.

Aerobic Exercise: The type of aerobic exercise in the present study will involve walking at moderate intensity. Walking is the primary mode of physical activity, given its widespread popularity and ease of administration to a broad segment of the elderly population [63]. We focus on moderate training, as studies indicate the greatest effects compared to light or vigorous exercise [64]. The frequency of aerobic exercise will amount to 30 min, 5 days per week. Moderate intensity is defined as 64–76% of the HRmax which is the most commonly used indicator for determining the intensity of aerobic exercise [4,11,56]. The maximum heart rate is calculated according to the type: HRmax = 220 − age.

Moderate intensity control will be monitored based on the participant’s Borg scale of subjective perception of fatigue during exercise by the caregiver at home. Specifically, exercise intensity is graded using a 6–20 category ratio scale (Borg Rating of Perceived Exertion (BRPE) Scale 6–20) according to fatigue and breathlessness. Medium volume is set to approximately 13 [56,65,66]. Caregivers will supervise all exercise sessions since these take place at home, whilst the sessions will be monitored by physiotherapists once a week for the first 2 weeks and subsequently once a month.

Resistance Exercise: The resistance exercise in the study will target the major muscle groups of the body and will be performed with limb weights at moderate intensity according to 50–69% of a maximum repetition (% 1-RM) [56]. The intervention will include 2 sets of 10 exercises of the main muscle groups starting with 8 repetitions which will progressively be increased to 12 repetitions. The frequency of resistance training will amount to three workouts per week (every 48 h), with a duration of 40–45 min per session, with a break of 1–3 min between sets [56,60,61,62,67,68]. Of the three exercise sessions, two will take place at the recruitment center under the supervision of the physiotherapist and one at home under the supervision of the caregiver. The moderate intensity control will additionally be monitored based on the scale Borg (Rating of Perceived Exertion). During the first two weeks, the physiotherapists will supervise the exercise at home once a week and subsequently once a month.

**Intervention group B:** Resistance exercise program

Intervention group B will perform the same resistance exercise program as intervention group A.

The aerobic exercise program and the resistance exercise program will both be followed progressively. Both intervention programs will also include warm-up and cool-down exercises lasting 5–10 min, respectively, with active exercises for the head, limbs, and torso, as well as stretching.

The interventions were selected and designed so as to provide the possibility of easy reproduction at home, promoting an active lifestyle which could be maintained even after the end of the study. Furthermore, the intervention program including illustrated instructions will be made available to the participants and must be followed for 12 weeks.

Strategies for monitoring and adhering to the intervention will comprise one (1) experienced physiotherapist’s visit to the patient’s home during the first two (2) weeks and then one (1) visit per month, one (1) phone call once a week during the first month, and then one (1) phone call per month. Participants and their caregivers will complete an exercise diary form.

Home visits and telephone communications are intended firstly to ensure that the exercises are performed correctly and safely according to the protocol, secondly to resolve any problems, and thirdly to encourage the participant’s effort.

**Control group C:** No participation in any exercise program

The control group participants will be encouraged to maintain their usual daily activity without engaging in any exercise program. Team members who deviate from their daily routine by following any physical exercise program will be excluded from the team.

## 5. Statistical Analysis

Descriptive and inferential statistical analysis will be carried out. The normality of the variables under consideration will be tested with the Shapiro–Wilk test. Possible dependencies between qualitative variables and categorical scales characteristics of the three different groups will be investigated using Pearson’s chi-squared test (χ^2^).

A paired t-test or the non-parametric Wilcoxon signed-rank test was performed to analyze whether there was any significant improvement in the cognitive function and mood (ACE-R, MMSE, TMT A-B, DST F-B, and GDS-15) and functionality (SFT, BBS, IADL, and IPAQ) after the subjects had completed the 12 weeks intervention of the two different exercise programs.

In order to investigate differences between groups, at baseline and after the intervention, one-way ANOVA F-tests, or Kruskal–Wallis tests for more than two independent samples will be carried out for all the mentioned variables, both at baseline and after the intervention (12 weeks). Bonferroni correction was applied to adjust for multiple pair-wise comparisons of the means for the main effects.

Pearson’s or Spearman’s correlation tests for the total changes from baseline will be applied accordingly to normality test. Variables correlated significantly will be used for further multivariate analysis. To further analyze the relationship between the changes of outcome measures, a multivariate linear regression analysis will also be applied for the total changes from baseline, in order to clarify the effect of exercise in cognitive function and functional performance. If group differences are observed at baseline, those variables are included as covariates in further analyses. To assess the effect on the primary and secondary outcome measures, analysis of covariance (ANCOVA) is used with cognitive domain scores on the post-tests as dependent variables, pre-test scores as covariates, and group (combined aerobic and resistance exercise program, single resistance exercise program, control) as the between-subject factor.

Statistical analyses will be carried out using the statistical software package SPSS 24.0 for Windows (SPSS Inc., Chicago, IL, USA). *p*-values less than 0.05 are considered statistically significant.

## 6. Discussion

The public health impact of Alzheimer’s disease is enormous [2,4,5]. Several studies have focused on the potential benefits of non-pharmacological approaches in patients with dementia [7]. In recent years, there is more interest in the role of physical exercise [8,9].

According to studies, it appears that systematic exercise, through a variety of mechanisms, can promote brain function and maintain and improve both cognitive functions and physical functions in elderly persons, as well as in persons with dementia [69,70,71,72].

Unfortunately, in various previous studies, there is no mention of the level of intensity, duration, and frequency of the exercise required [14,73,74,75], for an optimal exercise intervention in persons with Alzheimer’s disease. It is also possible that the benefits of exercise in the intervention groups, in terms of cognitive function, are greater in magnitude when compared to the control (no intervention) groups [73]. The use of a control group is essential to identify any differences between interventions [75]. Very few studies have supplemented data from the control group and no similar studies have been conducted in elderly adults with dementia (AD) [8].

There have been more studies in aerobic exercise and mixed interventions, while there have not been many studies regarding resistance interventions [9]. The comparison between resistance exercise on the one hand, and the combined intervention of both aerobic and resistance exercise on the other, has not been studied.

Findings regarding the cognitive benefits of exercise as a treatment for AD are inconsistent [10,11,14,71]. Inconsistencies in cognitive findings may be explained by methodological factors, such as differences in study samples and exercise parameters [10,11,14,71,73,74,75]. Moreover, another significant factor which has not been acknowledged as such, concerns inter-individual differences in exercise responses [10,11]. The methodology differences of the studies make it difficult to draw definitive conclusions about the optimal intervention in the cognitive and physical function for the optimal result, as regards the type of exercise, the duration, the frequency, and the intensity [10,11,14,71,73,74,75].

Exercise represents a viable, low-cost, personalized, and widely available, effective non-pharmacological treatment option in cognitive decline [64].

Several researchers suggest that interventions comprising home exercise programs are effective for older adults [76]. In addition, home exercise programs are cost-effective, and many older adults can benefit, since there are various limitations in accessing on-site programs in care centers [3,64,76].

## 7. Expected Results and Possible Benefits

Exercise seems to be promising for both prevention and improvement of cognitive–physical function as well as overall functioning, with evident effects on daily activities among both elderly and dementia patients.

This randomized controlled trial will contribute evidence on the potential effects of a supervised physical exercise program for persons with mild dementia (AD) living at home. Given that physical activity and exercise has a positive effect on health at any age, if the effect of exercise on persons with AD dementia is documented in our study, it could then complement medical treatment. It would also help physical therapists to design an individualized and appropriate exercise program for older persons with mild dementia in the community, in a way that can be implemented at home. In this context, regardless of results, there will be maximum objective benefit to public health.

## 8. Strengths and Limitations of This Study

- According to what we know, this randomized controlled trial (RCT) will be the first study to examine the effect of aerobic exercise combined with resistance exercise in comparison to resistance exercise only, in patients with dementia.
- It will also be the first study in Greece to examine the effect of aerobic exercise combined with resistance exercise in comparison to resistance exercise only in patients with dementia both in the center and at home.
- The intervention program is comprehensive and well-structured as well as easy to perform, without the necessity for specialized and expensive equipment.
- During the study, the COVID-19 restrictions created difficulties in the sample collection and, therefore, the duration of the study may have to be extended.

## Data Availability

Not applicable.
